# Risk prediction of severe chemotherapy-induced peripheral neuropathy in breast cancer patients receiving nab-paclitaxel: a clinical nomogram

**DOI:** 10.3389/fphar.2026.1794696

**Published:** 2026-09-08

**Authors:** Yujun Tong, Tiantian Liang, Jiangping Yu, Hong Ning, Xiaohong Zhang, Zhen Zhang, Siyu Liu, Min Yang

**Affiliations:** 1 Department of Breast Center, Mianyang Central Hospital, School of Medicine, University of Electronic Science and Technology of China, Mianyang, China; 2 Mianyang Central Hospital, School of Medicine, University of Electronic Science and Technology of China, Mianyang, China; 3 Department of Pharmacy, Mianyang Central Hospital, School of Medicine, University of Electronic Science and Technology of China, Mianyang, China; 4 Department of Thyroid and Breast Surgery, Guangyuan Central Hospital, Guangyuan, China

**Keywords:** body mass index, breast cancer, chemotherapy-induced peripheral neuropathy, diabetes, nab-paclitaxel, nomogram, risk prediction

## Abstract

**Importance:**

Chemotherapy-induced peripheral neuropathy (CIPN) is a frequent and dose-limiting toxicity of nab-paclitaxel in breast cancer patients. Severe CIPN can impair quality of life, limit treatment adherence, and lacks validated prediction tools for early risk stratification.

**Objective:**

To identify risk factors for grade ≥3 CIPN in breast cancer patients treated with nab-paclitaxel and to develop a predictive model for individualized risk assessment.

**Design:**

Retrospective cohort study using clinical data from January 2021 to January 2025. Predictors were selected with least absolute shrinkage and selection operator (LASSO) regression and included in multivariable logistic regression. A nomogram was constructed, and model performance was evaluated using area under the curve (AUC), Hosmer-Lemeshow test, calibration curve, and decision curve analysis. Restricted cubic spline analysis examined nonlinear associations of body mass index (BMI) and total bilirubin (TBIL) with CIPN risk.

**Setting:**

Mianyang Central Hospital, a tertiary referral center in China.

**Participants:**

A total of 403 breast cancer patients receiving nab-paclitaxel. Among them, 13.2% (53/403) developed CIPN ≥ Grade 3. Patients with severe CIPN more frequently had higher BMI, elevated TBIL, smoking or alcohol drinking, and diabetes, and lower platelet count, total protein, and hemoglobin (all *P* < 0.05).

**Main outcomes and measures:**

Occurrence of CIPN ≥ Grade 3 according to Common Terminology Criteria for Adverse Events (CTCAE) v5.0.

**Results:**

LASSO identified BMI, TBIL, smoking, alcohol drinking, and diabetes as key predictors. All were confirmed as independent risk factors in multivariable analysis, with diabetes showing the strongest association (adjusted OR = 4.01). The nomogram demonstrated good discrimination (AUC = 0.78), calibration (*P* = 0.898), and clinical utility. Restricted cubic spline analysis showed a linear association between TBIL and CIPN risk, and significantly increased risk with BMI ≥24 kg/m^2^.

**Conclusion and relevance:**

Elevated BMI, smoking, Alcohol drinking, diabetes, and TBIL are independent risk factors for severe CIPN in patients with breast cancer treated with nab-paclitaxel. The proposed nomogram may support early identification of high-risk patients and guide individualized prevention and management strategies.

## Highlights

- Question: Does chemotherapy-induced peripheral neuropathy (CIPN) in breast cancer patients is associated with factors such as age, anthropometrics, menopausal status, lifestyle factors, disease stage, surgical procedure, albumin-bound paclitaxel regimen, treatment cycles, or laboratory values?
- Findings: Elevated body mass index (BMI), smoking, alcohol drinking, diabetes, and total bilirubin (TBIL) are independent risk factors for severe CIPN in breast cancer patients receiving nab-paclitaxel. The predictive nomogram may aid early identification of high-risk patients and guide individualized preventive and therapeutic strategies.
- Meaning: The results of this study provide an evidence base for optimizing the safe and precise use of albumin-bound paclitaxel, offering valuable insights for both clinical management and treatment individualization in breast cancer.

## 1 Introduction

Breast cancer is the most common malignancy among women worldwide, posing a significant threat to female health (Bray et al., 2018). Current treatment strategies for breast cancer include surgery, chemotherapy, radiotherapy, endocrine therapy, targeted therapy, and immunotherapy. Although novel therapeutic targets continue to be discovered and new targeted agents developed, endocrine therapy strategies have been continuously optimized, and research on combination immunotherapy has advanced, systemic chemotherapy optimization and precision treatment remain critical clinical challenges.

Taxanes play a pivotal role in standard chemotherapy regimens for breast cancer and are classic cytotoxic agents. They inhibit tumor cell proliferation by disrupting microtubule dynamics (Weaver, 2014; Mekhail and Markman, 2002). However, Chemotherapy-induced peripheral neuropathy (CIPN) is a common and severe adverse effect of taxanes, characterized predominantly by sensory disturbances such as numbness, tingling, and burning sensations, typically distributed in a “glove-and-stocking” pattern in distal extremities (Park et al., 2013). CIPN significantly impairs daily life, increases the risk of falls and injuries, and has an incidence ranging from 68% to 84%, with over 30% of patients developing chronic CIPN, severely affecting quality of life and survival (Ibrahim and Ehrlich, 2020). Therefore, early identification and management of CIPN in breast cancer patients are of critical clinical importance.

Commonly used taxanes include paclitaxel, docetaxel, liposomal paclitaxel, and albumin-bound paclitaxel (nab-paclitaxel). Nab-paclitaxel employs nanotechnology to bind hydrophobic paclitaxel to albumin, enhancing tumor uptake and accumulation, thereby improving antitumor efficacy (Mittendorf et al., 2020). Compared with solvent-based paclitaxel, nab-paclitaxel is water-soluble, avoids hypersensitivity reactions, and does not require prophylactic anti-allergy treatment; however, it is more prone to inducing CIPN (Mittendorf et al., 2020). A meta-analysis by Lee Hwaryeon et al. reported that nab-paclitaxel exhibited superior objective response rate and disease control rate compared with solvent-based paclitaxel in patients with metastatic breast cancer, but CIPN incidence was higher (Lee et al., 2020). In contrast, a clinical study by Francesco Di Costanzo et al. indicated that nab-paclitaxel caused lower rates of neutropenia and that CIPN symptoms could improve relatively quickly (Di Costanzo et al., 2009). Overall, nab-paclitaxel remains one of the most effective chemotherapeutic agents for breast cancer, yet the risk of CIPN cannot be overlooked.

Currently, only duloxetine has shown evidence supporting its use for painful CIPN (Loprinzi et al., 2020), and effective preventive strategies remain limited. Thus, investigating risk factors for CIPN has become a research focus. Known risk factors include cumulative dose (Liang et al., 2024), age ≥45 years (Barginear et al., 2019), and BMI (Ghoreishi et al., 2018); however, potential contributors such as chemotherapy duration, functional status, liver function, anemia, physical condition, and psychological factors have been insufficiently explored. With the advancement of precision medicine, developing risk assessment models to guide individualized treatment strategies is a promising future direction. Nevertheless, predictive models for CIPN induced by nab-paclitaxel in breast cancer patients are scarce.

Therefore, this study aims to identify risk factors associated with CIPN in breast cancer patients receiving nab-paclitaxel chemotherapy and to develop a predictive model, which may provide a scientific basis for early identification and individualized intervention in high-risk patients, with significant clinical and research implications.

## 2 Materials and methods

### 2.1 Data source and collection

**FIGURE 1:**
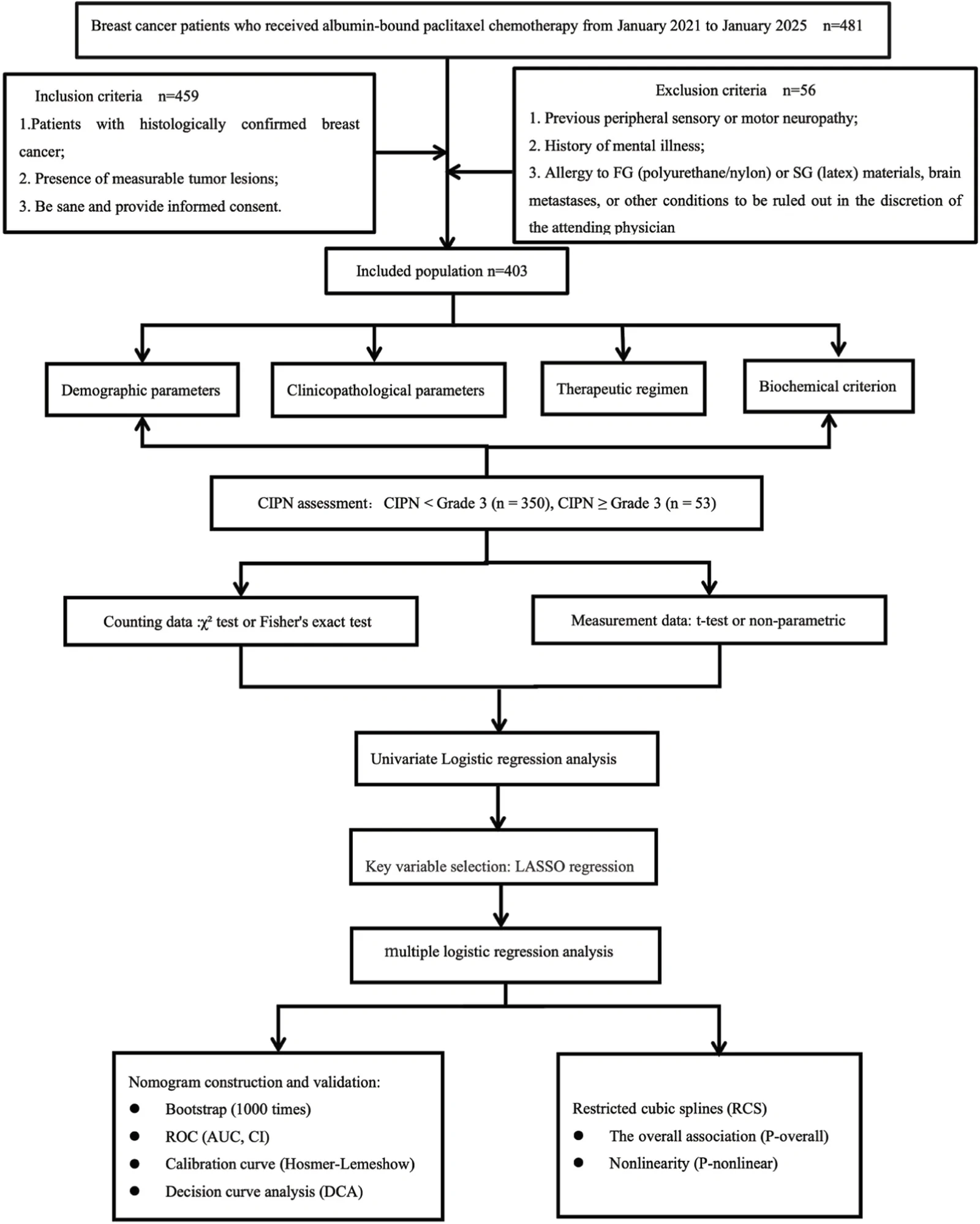
Participant flowchart and the research design.

This retrospective study utilized medical records from Mianyang Central Hospital. Clinical data of breast cancer patients who received albumin-bound paclitaxel chemotherapy between January 2021 and January 2025 were collected, including demographic characteristics, clinical and pathological parameters, treatment details, and laboratory indicators previously reported as potential risk factors for CIPN (Figure 1). Specifically, data included: (1) demographics: age, height, weight, BMI, menstrual status, smoking, and alcohol drinking; (2) clinical and pathological parameters: tumor stage and subtype, distant metastasis, and lymph node involvement; (3) treatment details: chemotherapy regimen and drug dose; (4) biochemical indicators: complete blood count and liver and renal function parameters. All data were anonymized to ensure patient confidentiality. This study (ethical approval number: S20250250-01) was approved by the Mianyang Central Hospital, School of Medicine, University of Electronic Science and Technology of China, and all protocols were performed under the guidelines of the Helsinki Declaration (World Medical Association Declaration of Helsinki: ethical principles for medical research involving human subjects, 2013).

### 2.2 Inclusion and exclusion criteria

Inclusion criteria:

1. Histologically confirmed breast cancer;
2. Received at least one cycle of nab-paclitaxel chemotherapy at our institution;
3. Complete clinical records and available CTCAE-based CIPN assessment data.

Exclusion criteria:

1. Pre-existing peripheral sensory or motor neuropathy;
2. History of psychiatric disorders;
3. Brain metastases or other conditions that could interfere with reliable neurological assessment, as determined by the treating physician.

### 2.3 CIPN assessment

CIPN was assessed according to the National Cancer Institute Common Terminology Criteria for Adverse Events (CTCAE), version 5.0. All evaluations were performed by uniformly trained clinical pharmacists or specialized oncology nurses who had completed standardized training in the CTCAE 5.0 neurotoxicity module, thereby ensuring the consistency and comparability of assessments.

CIPN was graded on a five-point scale (grades 1–5), where grade 1 indicates mild symptoms without functional impairment and grade 5 indicates death related to neurotoxicity (Supplementary Table S1). For analytical purposes, the outcomes were further categorized into two groups: CIPN < grade 3 (grades 1–2), defined as mild to moderate neurotoxicity with preserved self-care ability, and CIPN ≥ grade 3 (grades 3–5), defined as severe to life-threatening neurotoxicity characterized by marked functional impairment or loss of self-care capacity. Among the grades, grade 5 is defined as death resulting from neurotoxicity. According to drug safety guidelines, patients experiencing grade 3 events required treatment interruption until recovery to ≤ grade 1, while grade 4 events mandated permanent discontinuation of therapy. For grade 5, the case should be included in the statistical analysis of adverse event-related deaths.

### 2.4 Statistical analysis

Analyses were performed using Python 3.9 and R 4.0.5. Patients were stratified into non-severe and severe CIPN groups. Continuous variables were assessed for normality (Shapiro–Wilk test) and homogeneity of variance (Levene’s test). Parametric data were compared using independent-sample t-tests, and non-parametric data with Mann–Whitney U tests. Categorical variables were analyzed using χ^2^ or Fisher’s exact tests as appropriate. As this was a retrospective study, no *a priori* sample size calculation was performed. The final multivariable model included five predictors and 53 outcome events, yielding an events-per-variable (EPV) ratio of 10.6, which met the recommended criterion for logistic regression modeling.

Feature selection was conducted using LASSO regression (glmnet package) with L1 regularization; optimal λ was determined by five-fold cross-validation. Variables with *P* < 0.05 in univariate logistic regression were further included in multivariate logistic regression via a stepwise approach. Nonlinear relationships between predictors and CIPN risk were explored using restricted cubic splines (RCS, rms package) with four fixed knots, with overall association (P-overall) and nonlinearity (P-nonlinear) assessed via likelihood ratio tests.

Model performance was evaluated using: (1) nomograms to visualize predictor contributions; (2) receiver operating characteristic (ROC) curves with area under the curve (AUC) and 95% confidence intervals (pROC package); (3) decision curve analysis (rmda package); and (4) calibration curves with Hosmer–Lemeshow goodness-of-fit test (*P* > 0.05 indicating adequate calibration). All tests were two-sided with a significance threshold of α = 0.05.

## 3 Results

### 3.1 Baseline characteristics

A total of 403 patients were included, comprising 350 with CIPN < Grade 3 and 53 with CIPN ≥ Grade 3. The median age of the cohort was 52.0 years (IQR 45.0–60.0), and no statistically significant intergroup difference was observed (*P* = 0.790). A significantly higher BMI (median 25.0 vs. 23.3 kg/m^2^, *P* = 0.002) was documented in the CIPN ≥ Grade 3 group. Furthermore, elevated prevalences of smoking (24.5% vs. 6.6%, *P* < 0.001), alcohol drinking (37.7% vs. 12.9%, *P* < 0.001), and diabetes (24.5% vs. 6.6%, *P* < 0.001) were identified in this patient subset. The menopausal status distribution (62.3% vs. 59.7%, *P* = 0.724) and the prevalence of hypertension (18.9% vs. 10.9%, *P* = 0.093) were found to be comparable between the groups. Detailed baseline characteristics for all variables, including non-significant ones, were provided in Table 1 and Supplementary Table S1.

**TABLE 1:** Baseline characteristics of breast cancer patients treated with nab-paclitaxel, stratified by CIPN severity.

Variables	Total (n = 403)	CIPN < grade 3 (n = 350)	CIPN ≥ grade 3 (n = 53)	Test statistic	P Value
Demographic characteristics					
Age, median (Q_1_–Q_3_), years	52.0 (45.0–60.0)	52.0 (45.3–60.0)	52.0 (45.0–63.0)	*Z* = −0.27	0.790
BMI, median (Q_1_–Q_3_), kg/m^2^	23.4 (22.0–25.2)	23.3 (22.0–25.0)	25.0 (23.0–26.6)	*Z* = −3.14	0.002
Menopause, n (%)	242 (60.1)	209 (59.7)	33 (62.3)	*χ* ^2^ = 0.12	0.724
Smoking, n (%)	36 (8.9)	23 (6.6)	13 (24.5)	*χ* ^2^ = 16.10	<0.001
Alcohol drinking, n (%)	65 (16.1)	45 (12.9)	20 (37.7)	*χ* ^2^ = 21.06	<0.001
Hypertension, n (%)	48 (11.9)	38 (10.9)	10 (18.9)	*χ* ^2^ = 2.82	0.093
Diabetes, n (%)	36 (8.9)	23 (6.6)	13 (24.5)	*χ* ^2^ = 16.10	<0.001
Tumor characteristics					
Tumor size, median (Q_1_–Q_3_), cm	2.0 (2.0–3.0)	2.0 (2.0–3.0)	2.0 (2.0–3.0)	*Z* = −1.05	0.294
Tumor subtype, n (%)	​	​	​	*χ* ^2^ = 5.81	0.121
• Luminal A	109 (27.1)	98 (28.0)	11 (20.8)	​	​
• Luminal B	125 (31.0)	103 (29.4)	22 (41.5)	​	​
• HER2+	80 (19.9)	74 (21.1)	6 (11.3)	​	​
• TNBC	89 (22.1)	75 (21.4)	14 (26.4)	​	​
Tumor stage, n (%)	​	​	​	–	0.171
• I	12 (3.0)	10 (2.9)	2 (3.8)	​	​
• II	266 (66.0)	229 (65.4)	37 (69.8)	​	​
• III	110 (27.3)	100 (28.6)	10 (18.9)	​	​
• IV	15 (3.7)	11 (3.1)	4 (7.6)	​	​
LN metastasis, n (%)	313 (77.7)	271 (77.4)	42 (79.3)	*χ* ^2^ = 0.09	0.767
Distant metastasis, n (%)	15 (3.7)	11 (3.1)	4 (7.6)	*χ* ^2^ = 1.41	0.234
Treatment characteristics					
Neo-adjuvant therapy, n (%)	214 (53.1)	191 (54.6)	23 (43.4)	*χ* ^2^ = 3.97	0.137
Adjuvant therapy, n (%)	174 (43.2)	148 (42.3)	26 (49.1)	​	​
Palliative therapy, n (%)	15 (3.7)	11 (3.1)	4 (7.6)	​	​
Chemotherapy dose, n (%)	​	​	​	*χ* ^2^ = 0.00	0.989
• Conventional	281 (69.7)	244 (69.7)	37 (69.8)	​	​
• Dense	122 (30.3)	106 (30.3)	16 (30.2)	​	​
Cumulative dose, median (Q_1_–Q_3_), mg	1897 (1,697–2,328)	1887 (1,689–2,332)	1978 (1717–2,309)	Z = −0.514	0.607
TP, mean ± SD, g/L	71.8 ± 5.3	72.0 ± 5.3	70.3 ± 5.7	*t* = 2.25	0.025
Hb, mean ± SD, g/L	126.2 ± 13.5	126.8 ± 13.4	122.2 ± 13.8	*t* = 2.32	0.021
PLT, median (Q_1_–Q_3_), ×10^9^/L	220.3 (182.1–257.0)	224.2 (188.0–259.5)	193.9 (162.6–237.7)	*Z* = −3.11	0.002
TBIL, median (Q_1_–Q_3_), μmol/L	10.6 (7.5–13.7)	10.4 (6.9–13.3)	12.3 (8.8–14.6)	*Z* = −2.47	0.014
Other laboratory parameters	—	—	—	—	>0.05

Abbreviations: t, t-test; Z, Mann-Whitney test; χ^2^, Chi-square test; -, fisher exact; SD, standard deviation; M, median; Q_1_, 1st Quartile; Q_3_, 3st Quartile; CIPN, chemotherapy-induced peripheral neuropathy; BMI, body mass index; LN, lymph node; TP, total protein; Hb, hemoglobin; PLT, platelet count; TBIL, total bilirubin.

### 3.2 Clinical and laboratory parameters

No statistically significant intergroup differences were observed in tumor size (median 2.0 cm), stage distribution, or metastasis rates (all *P* > 0.05). Treatment-related variables such as neoadjuvant chemotherapy use (43.4% vs. 54.6%, *P* = 0.137) and conventional dosing intensity (69.8% in both groups) were comparable. Significantly lower levels of total protein (70.3 ± 5.7 vs. 72.0 ± 5.3 g/L, *P* = 0.025), hemoglobin (122.2 ± 13.8 vs. 126.8 ± 13.4 g/L, *P* = 0.021), and platelet counts (median 193.9 vs. 224.2 × 10^9^/L, *P* = 0.002) were demonstrated in the CIPN ≥ Grade 3 group, and a significantly higher total bilirubin level (median 12.3 vs. 10.4 μmol/L, *P* = 0.014) was concurrently identified. No significant differences were identified in the remaining hematologic, hepatic, or renal parameters; detailed data were provided in Supplementary Table S2.

### 3.3 LASSO regression for key variable selection

**FIGURE 2:**
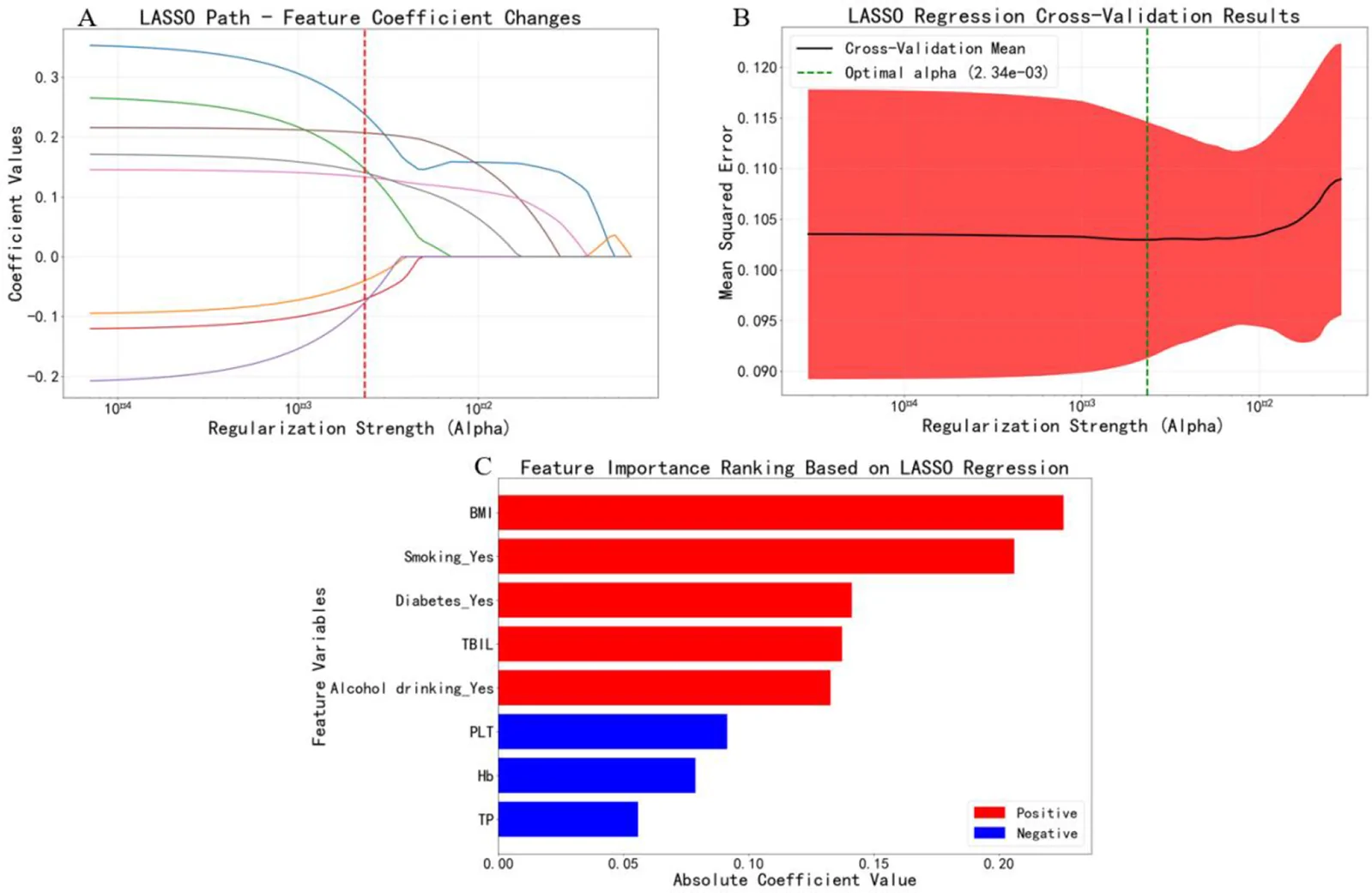
LASSO regression for important variable selection. **(A,B)** show the LASSO coefficient path and cross-validation curve. **(A)** shows the LASSO coefficient path; the red vertical line indicates the optimal λ value. **(B)** shows the cross-validation curve, with the optimal λ (λ = 0.002) marked by a green dashed line. **(C)** shows the feature importance ranking of the variables selected by LASSO.

Eight baseline variables that demonstrated significant intergroup differences were entered into a LASSO regression analysis. The optimal penalty parameter (λ = 0.002) was selected via five-fold cross-validation (Figure 2). BMI (β = +2.449), total bilirubin (TBIL; β = +1.909), smoking history (β = +1.255), diabetes (β = +1.090), and alcohol drinking (β = +1.075) were identified as positive predictors, whereas platelet count (β = −2.321), total protein (β = −1.609), and hemoglobin (β = −1.308) were identified as negative predictors. Among these, the strongest predictive effects were observed for BMI and platelet count.

### 3.4 Variable selection for nomogram construction

**FIGURE 3:**
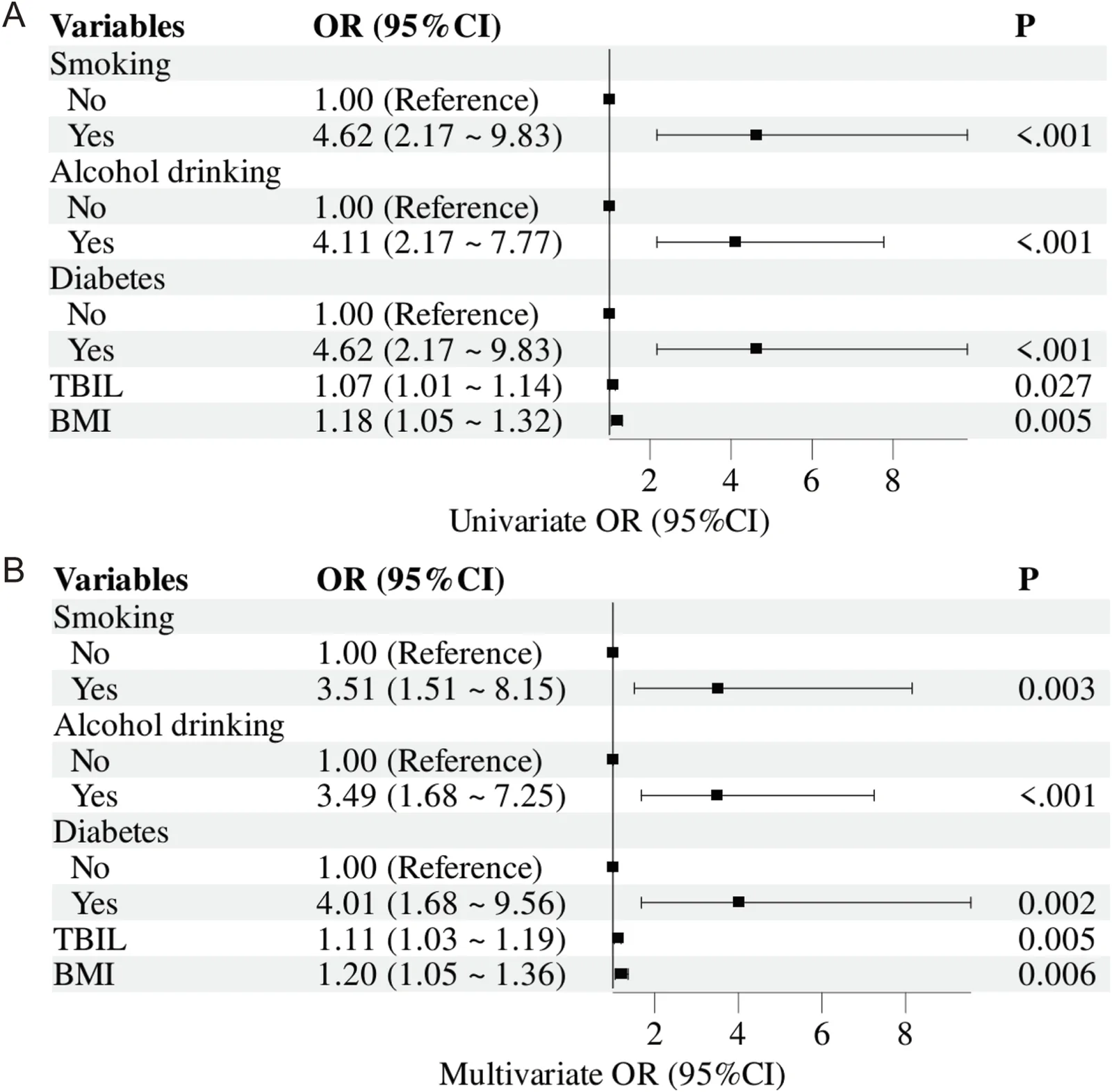
Forest plots of univariate and multivariate analyses. **(A,B)** represent univariate and multivariate forest plots, respectively.

Based on the key predictors identified by LASSO regression (BMI, PLT, TBIL, TP, Hb, smoking, diabetes, and alcohol drinking), we performed binary logistic regression to evaluate their associations with CIPN ≥ Grade 3 risk. Given the limited number of CIPN ≥ Grade 3 cases (n = 53), we prioritized the top five most influential variables from the LASSO analysis (BMI, TBIL, alcohol drinking, smoking, and diabetes) to ensure statistical power (10–20 events per variable). As shown in Table 2 and Figure 3, univariate analysis demonstrated that smoking (OR = 4.62, 95% CI: 2.17–9.83), alcohol drinking (OR = 4.11, 95% CI: 2.17–7.77), diabetes (OR = 4.62, 95% CI: 2.17–9.83), elevated TBIL (OR = 1.07 per unit increase, 95% CI: 1.01–1.14), and higher BMI (OR = 1.18 per unit increase, 95% CI: 1.05–1.32) were significantly associated with CIPN ≥ Grade 3 development (all P < 0.05).

**TABLE 2:** Univariate and multivariate logistic regression analysis of risk factors for CIPN ≥ Grade 3.

Risk factors	Univariate		Multivariate	
	OR (95% CI)	P	OR (95% CI)	P
Smoking				
No	1.00 (References)	​	1.00 (References)	​
Yes	4.62 (2.17–9.83)	**<0.001**	3.51 (1.51–8.15)	**0.003**
Alcohol drinking				
No	1.00 (References)	​	1.00 (References)	​
Yes	4.11 (2.17–7.77)	**<0.001**	3.49 (1.68–7.25)	**<0.001**
Diabetes				
No	1.00 (References)	​	1.00 (References)	​
Yes	4.62 (2.17–9.83)	**<0.001**	4.01 (1.68–9.56)	**0.002**
TBIL	1.07 (1.01–1.14)	**0.027**	1.11 (1.03–1.19)	**0.005**
BMI	1.18 (1.05–1.32)	**0.005**	1.20 (1.05–1.36)	**0.006**

Bold values indicate statistically significant associations (P < 0.05).

In multivariate analysis, smoking (aOR = 3.51, 95% CI: 1.51–8.15), alcohol drinking (aOR = 3.49, 95% CI: 1.68–7.25), diabetes (aOR = 4.01, 95% CI: 1.68–9.56), TBIL (aOR = 1.11 per unit increase, 95% CI: 1.03–1.19), and BMI (aOR = 1.20 per unit increase, 95% CI: 1.05–1.36) were confirmed as independent risk factors for CIPN ≥ Grade 3 (all P < 0.01). The strongest effect size was exhibited by diabetes (aOR = 4.01); a 4-fold higher CIPN ≥ Grade 3 risk was estimated for diabetic patients compared with non-diabetic patients. Each 1-unit increase in BMI and TBIL was associated with 20% and 11% elevated CIPN ≥ Grade 3 risk, respectively. Comparable risk magnitudes were observed for smoking and alcohol drinking (aOR ≈3.5), and it was suggested that lifestyle factors contributed significantly to CIPN ≥ Grade 3 susceptibility, independent of metabolic and hematological parameters.

### 3.5 Nomogram construction and validation

**FIGURE 4:**
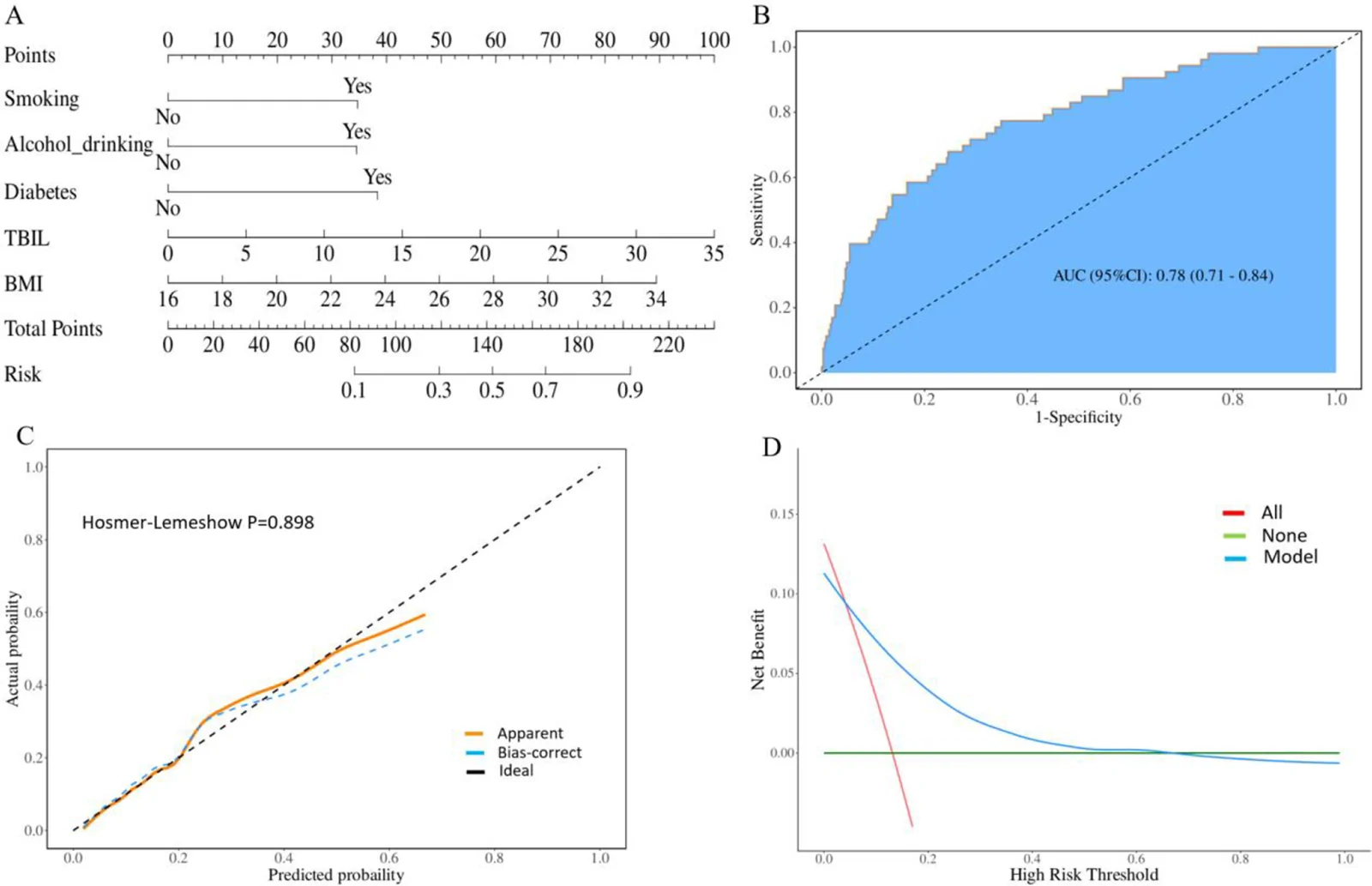
Nomogram construction and validation. **(A)** Nomogram for predicting CIPN ≥ Grade 3, incorporating three categorical variables (smoking status, alcohol consumption, and diabetes) and two continuous variables (TBIL and BMI). Each predictor is assigned points, and the total points correspond to the predicted risk. **(B)** ROC curve demonstrating favorable discriminative ability (AUC = 0.78). **(C)** Calibration curve showing close agreement between predicted and observed CIPN ≥ Grade 3 probabilities. **(D)** Decision curve analysis evaluating the net benefit of the nomogram across threshold probabilities, where: Model line: Net benefit of treatment decisions based on the prediction model. All line: Theoretical benefit of treating all patients. None line: Baseline of no interventions. Threshold probability: The minimum risk level at which clinicians would recommend treatment. Net benefit: Calculated as (true positives - weighted false positives)/total patients, with weighting derived from threshold probabilities.

In this study, a CIPN ≥ Grade 3 risk prediction nomogram (Figure 4A) was constructed based on significant factors identified by multivariate logistic regression. Internal validation was performed using the bootstrap method with 1,000 resamples to evaluate model performance. The Hosmer-Lemeshow test indicated excellent model fit (P = 0.898). Discriminative ability was assessed via ROC analysis (Figure 4B), yielding an AUC of 0.78 (95% CI: 0.71–0.84), suggesting good performance in the internal dataset. Furthermore, strong agreement between predicted and observed CIPN ≥ Grade 3 probabilities was demonstrated by calibration curves (Figure 4C), and model reliability was supported.

Decision curve analysis (DCA) was employed to evaluate clinical utility across risk thresholds. Superior net benefit within the 0.1–0.6 probability range was demonstrated by the model compared with the “All” or “None” strategies (Figure 4D). At thresholds below 0.1, the “All” strategy was shown to be preferable, whereas at thresholds exceeding 0.6, greater net benefit was provided by the “None” strategy. Notably, a high positive predictive value (PPV = 0.94; Table 3) was obtained, and the model’s potential for clinical decision-making in CIPN ≥ Grade 3 risk stratification was thereby underscored.

**TABLE 3:** Model performance metrics.

AUC (95% CI)	Accuracy (95% CI)	Sensitivity (95% CI)	Specificity (95% CI)	PPV (95% CI)	NPV (95% CI)	Cut off
0.78	0.74	0.75	0.68	0.94	0.29	0.137
(0.71–0.84)	(0.70–0.78)	(0.71–0.80)	(0.55–0.80)	(0.91–0.97)	(0.21–0.37)	

### 3.6 Nonlinear association analysis using restricted cubic splines

**FIGURE 5:**
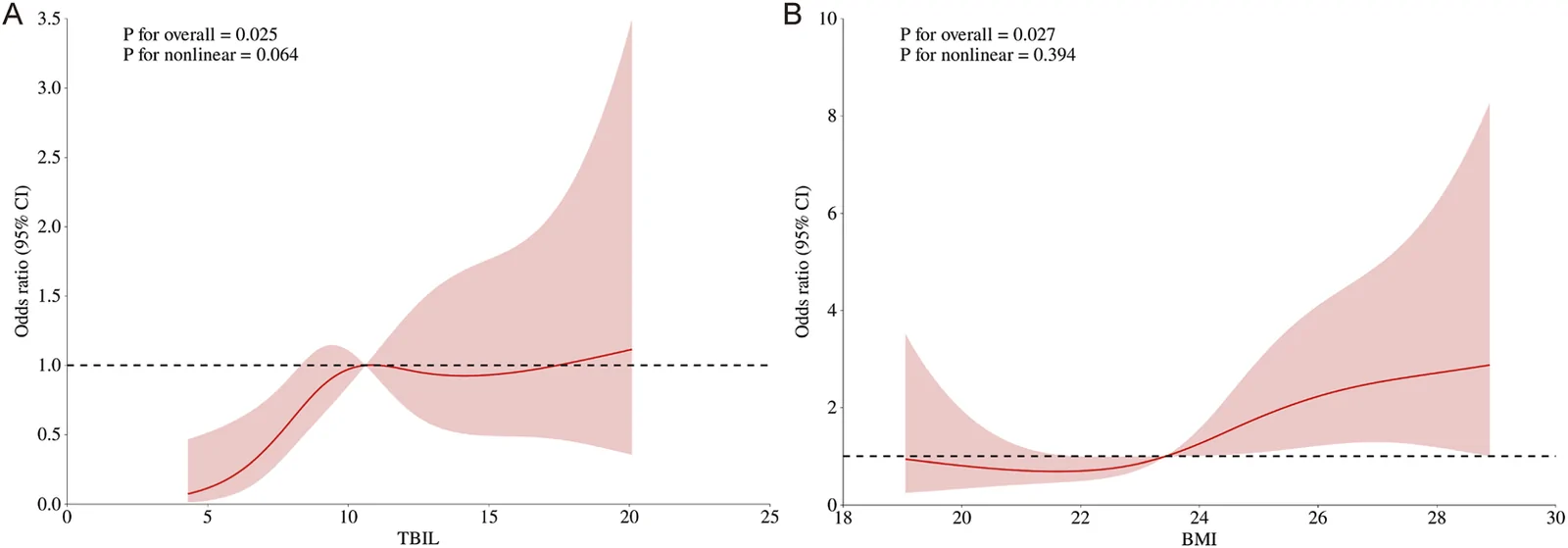
Restricted cubic spline (RCS) analyses. **(A)** The RCS plot for TBIL was adjusted for covariates including smoking status, alcohol drinking, diabetes, and BMI. **(B)** The RCS plot for BMI was adjusted for covariates including smoking status, alcohol drinking, diabetes, and TBIL.

Restricted cubic spline (RCS) models were used to examine potential nonlinear relationships between the predictors (TBIL and BMI) and the outcome, with adjustment for relevant covariates. For TBIL, a statistically significant overall association was observed (*P* for overall = 0.025). A protective effect (OR < 1) was demonstrated by the spline curve at TBIL levels below 11 μmol/L, and a gradual increase in OR values was observed as TBIL levels increased. However, no significant nonlinearity was detected (P for nonlinear = 0.064) (Figure 5A). At TBIL concentrations exceeding 11 μmol/L, the OR estimates stabilized around the null value (OR ≈ 1). In contrast, a significant overall association with the outcome was shown for BMI (P for overall = 0.027), this association was found to follow a predominantly linear pattern (P for nonlinear = 0.394) (Figure 5B). Notably, increased risk (OR > 1) was associated with elevated BMI levels (≥24 kg/m^2^), and most confidence intervals excluded the null value. Conversely, point estimates below 1 (OR < 1) were exhibited for lower BMI levels (<23.6 kg/m^2^); however, the confidence intervals consistently included unity.

## 4 Discussion

This study aimed to develop a risk prediction model for CIPN in breast cancer patients treated with nab-paclitaxel, based on real-world clinical data. To our knowledge, this is the first study to systematically integrate demographic, clinical, and biochemical indicators into a predictive framework for individualized risk stratification. The main findings are as follows: (1) patients with severe CIPN (Grade ≥3) exhibited higher BMI and higher prevalence of smoking, alcohol drinking, and diabetes, along with lower platelet counts, total protein, and hemoglobin, and elevated total bilirubin; (2) multivariate logistic regression identified smoking (aOR = 3.51), alcohol drinking (aOR = 3.49), diabetes (aOR = 4.01), total bilirubin (aOR = 1.11 per μmol/L), and BMI (aOR = 1.20 per kg/m^2^) as independent predictors, with diabetes exerting the strongest effect; (3) the constructed nomogram demonstrated good discrimination (AUC = 0.78), calibration, and net clinical benefit; and (4) restricted cubic spline analysis revealed a linear risk association for bilirubin and a marked increase in risk at BMI ≥24 kg/m^2^. Collectively, these findings confirm previously reported risk factors while highlighting the potential role of metabolic and lifestyle-related parameters in CIPN development.

### 4.1 Pathophysiological mechanisms of nab-paclitaxel–induced neuropathy

Nab-paclitaxel, a potent microtubule-stabilizing agent, induces neurotoxicity through several interconnected mechanisms. Its primary action involves disruption of microtubule dynamics, leading to impaired axonal transport of essential cargoes such as vesicles, mitochondria, and proteins (Wiranata et al., 2024; Klein and Lehmann, 2021). This dysfunction disproportionately affects long sensory axons in the dorsal root ganglia (DRG), rendering them particularly vulnerable (Klein and Lehmann, 2021).

In addition, nab-paclitaxel induces mitochondrial injury—manifested as swelling, cristae disruption, and increased permeability transition pore opening—resulting in impaired ATP synthesis, calcium dysregulation, and excessive reactive oxygen species (ROS) generation (Starobova and Vetter, 2017; Gornstein and Schwarz, 2014). These processes culminate in oxidative injury to DNA, proteins, and lipids (Starobova and Vetter, 2017). Furthermore, activation of microglia and satellite glial cells promotes release of pro-inflammatory cytokines (e.g., IL-1β, TNF-α), exacerbating neuroinflammation and neuropathic pain (Byrd-Leifer et al., 2001). These mechanisms collectively underlie the pathogenesis of nab-paclitaxel–induced neuropathy.

### 4.2 Lifestyle and metabolic risk factors

A key strength of this study lies in identifying lifestyle and metabolic conditions as major contributors to severe CIPN. These factors likely amplify the neurotoxic burden of nab-paclitaxel through synergistic mechanisms.

#### 4.2.1 Smoking and alcohol drinking

Our results confirmed that smoking and alcohol drinking are independent risk factors for severe CIPN, consistent with previous reports (Chopra and Tiwari, 2012). Smoking induces chronic systemic inflammation and oxidative stress, placing the nervous system in a “pre-injured” state (Sadowski and Houck, 2022). Toxic compounds and free radicals in cigarette smoke continuously activate immune responses, depleting neuronal and glial antioxidant defenses, thereby reducing tolerance to paclitaxel neurotoxicity (Khanna et al., 2013). Additionally, smoking-related microvascular dysfunction impairs peripheral nerve perfusion, causing ischemia and hypoxia that exacerbate axonal injury and delay repair (Julian et al., 2019).

Similarly, chronic alcohol intake directly damages neurons and Schwann cells, leading to axonal degeneration and demyelination (Sadowski and Houck, 2022). Alcohol abuse is often accompanied by nutritional deficiencies, particularly thiamine (vitamin B1) deficiency, which aggravates mitochondrial dysfunction and increases neuronal vulnerability under paclitaxel-induced metabolic stress (Sivandzade et al., 2020). Although nab-paclitaxel formulations do not contain ethanol, long-term alcohol drinking creates a pre-existing inflammatory and neurotoxic milieu that sensitizes the nervous system to chemotherapy, accelerating CIPN onset and progression.

#### 4.2.2 Diabetes

Diabetes emerged as the strongest risk factor (aOR = 4.01), consistent with previous studies (Li et al., 2023; Yang et al., 2022). Chronic hyperglycemia induces oxidative and inflammatory injury through pathways such as polyol flux, advanced glycation end-products, and protein kinase C activation (Pop-Busui et al., 2017; Jung et al., 2025). Consequently, peripheral nerves in diabetic patients exist in a pre-injured state, lowering tolerance to nab-paclitaxel toxicity. Diabetes-related microvascular dysfunction further impairs neural repair and prolongs recovery (Feldman et al., 2017).

#### 4.2.3 Body mass index

In this study, higher BMI was significantly associated with increased risk of CIPN, particularly at BMI ≥24 kg/m^2^ (aOR = 1.20), consistent with prior studies (Wiranata et al., 2024; Timmins et al., 2022). Mechanistically, obesity alters pharmacokinetics of lipophilic agents, prolonging peripheral nerve exposure, and promotes chronic low-grade inflammation (elevated TNF-α, IL-6), leading to glial activation, demyelination, and axonal degeneration (Verbraecken et al., 2006; Adriaenssens et al., 2025; Yanbing et al., 2025). Metabolic and microvascular disturbances, including insulin resistance and microvascular dysfunction, further impair neural perfusion and repair (Wiranata et al., 2024; Muris et al., 2013). Clinically, each 1 kg/m^2^ BMI increase was associated with higher CIPN risk (OR 1.13), and meta-analytic data suggest a 15% higher relative risk per 5 kg/m^2^ increase (Yanbing et al., 2025). These findings underscore BMI as a modifiable, dose-dependent risk factor. High-BMI patients may benefit from individualized dose adjustments, early neurological monitoring, and lifestyle interventions to mitigate CIPN risk.

#### 4.2.4 Total bilirubin

In this study, total bilirubin (TBIL) levels were significantly associated with the risk of severe CIPN, with each 1 μmol/L increase corresponding to an 11% higher risk (aOR = 1.11, 95% CI: 1.03–1.19, *P* < 0.01). Restricted cubic spline analysis suggested a biphasic effect, where low TBIL levels may confer protection, whereas elevated levels increase risk, indicating that TBIL may serve as a metabolic stress marker and a potential predictive factor for CIPN. Recent evidence has linked chemotherapy-induced neurotoxicity to oxidative stress and inflammatory responses (Starobova and Vetter, 2017; Gupta et al., 2022). TBIL represents a dual signal of endogenous antioxidant capacity and metabolic stress. Within physiological ranges, mild elevations in TBIL exert antioxidant effects by scavenging free radicals, inhibiting lipid peroxidation, and modulating immune responses, thereby providing neuroprotective buffering. However, excessive TBIL elevation—often reflecting impaired hepatic metabolism or cholestasis—may coincide with delayed drug clearance, prolonged exposure to active metabolites, and increased systemic oxidative and inflammatory burden, collectively promoting neurotoxicity. This dual role is consistent with prior evidence demonstrating that bilirubin can have both protective and detrimental effects depending on its level (Bellarosa and Tiribelli, 2022; Vitek et al., 2023; Pranty et al., 2022).

Clinical studies further support this concept. Lower bilirubin levels have been associated with increased risk of diabetic peripheral neuropathy, suggesting that insufficient antioxidant capacity may compromise neural repair (Li et al., 2023; Abe et al., 2021). Additionally, impaired liver function during chemotherapy has been reported as a risk factor for paclitaxel-induced CIPN (Lixian et al., 2024). Our findings are thus in line with prior evidence, supporting the notion that TBIL may serve both as a risk marker and a mechanistic indicator of oxidative stress and metabolic burden in CIPN development. Future studies should explore the nonlinear effects of TBIL and clarify its utility in risk stratification and individualized chemotherapy management.

These results highlight the importance of pre-treatment risk assessment and patient counseling, particularly regarding lifestyle modifications such as smoking cessation and moderate alcohol intake. Active management of comorbidities, including strict glycemic control in diabetes, along with potential antioxidant and anti-inflammatory interventions, may be crucial to mitigate CIPN severity. For instance, GLP-1 receptor agonists have been shown to alleviate paclitaxel-induced neurotoxicity by reducing oxidative stress and neuroinflammation, further supporting strategies that target diabetes-related pathophysiology to prevent or lessen CIPN (Jung et al., 2025).

### 4.3 Limitations and future directions

Several limitations should be acknowledged. First, as a retrospective real-world study, residual bias due to incomplete clinical information and unmeasured confounders cannot be excluded. Second, the single-center design and limited number of severe CIPN cases (n = 53) may restrict generalizability. Third, the potential “coasting effect”—progression of neuropathy after treatment completion—was not evaluated.

Notably, although diabetes was identified as one of the most important independent risk factors in this study, due to the limitations of retrospective data collection, information regarding diabetes-related treatments in some patients (types of hypoglycemic agents, duration of use, and dosage) was incompletely recorded, resulting in a certain degree of missing data. Therefore, further analysis of different antidiabetic treatment regimens could not be performed. In addition, some patients may have received other anticancer or non-oncologic treatments; however, related treatment information was also incompletely documented, and substantial heterogeneity existed among different therapeutic regimens. Owing to limitations in sample size and data completeness, we were unable to further evaluate the potential impact of these concomitant treatments on the risk of CIPN.

Furthermore, all patients in this study received albumin-bound paclitaxel (nab-paclitaxel). Therefore, whether the risk prediction model and associated risk factors established in this study are also applicable to patients treated with conventional paclitaxel remains to be further validated. Previous studies have shown that increasing age, higher cumulative drug dose, hypertension, and elevated BMI are associated with an increased risk of paclitaxel-induced peripheral neuropathy, which is generally consistent with some of the findings observed in our study (Lixian et al., 2024; Hiramoto et al., 2021). However, due to differences between albumin-bound paclitaxel and conventional paclitaxel in formulation composition, drug delivery methods, pharmacokinetic characteristics, and toxicity profiles, the magnitude of these risk factors and the applicability of the predictive model may not be fully equivalent between the two regimens.

In the future, multicenter and large-scale prospective studies are warranted to externally validate the predictive model developed in this study. These studies should systematically collect comprehensive information on prior and concurrent treatments, diabetes management, and long-term follow-up data to further clarify the effects of various clinical factors on the occurrence and progression of CIPN. Meanwhile, mechanistic studies are needed to elucidate the biological links between diabetes, obesity, bilirubin metabolism abnormalities, lifestyle factors, and albumin-bound paclitaxel-induced neurotoxicity, and to further verify the applicability of these risk factors in patients receiving conventional paclitaxel.

Despite these limitations, the nomogram developed in this study still provides a simple and clinically practical risk assessment tool with good predictive performance, which may facilitate the identification of high-risk patients prior to chemotherapy and support more targeted monitoring, intervention, and individualized treatment strategies.

## 5 Conclusion

In this real-world cohort of 403 breast cancer patients treated with nab-paclitaxel, 13.2% developed severe CIPN. Elevated BMI, smoking, alcohol drinking, diabetes, and higher total bilirubin were identified as independent predictors, with diabetes exerting the strongest effect. The proposed nomogram integrating these variables showed robust discrimination, calibration, and clinical utility, offering a practical tool for individualized risk prediction. RCS analysis further revealed a linear association with total bilirubin and a markedly increased risk at BMI ≥24 kg/m^2^. These findings highlight that readily available clinical and biochemical indicators can facilitate early identification of high-risk patients, support tailored dosing strategies, and improve the safety of nab-paclitaxel therapy. Future multicenter, prospective validation and mechanistic studies are warranted to strengthen the external generalizability of this model and to clarify the biological links between metabolic factors and CIPN, ultimately paving the way for clinical translation.

## Data Availability

The raw data supporting the conclusions of this article will be made available by the authors, without undue reservation.
