# Supplementary material for: Risk prediction of severe chemotherapy-induced peripheral neuropathy in breast cancer patients receiving nab-paclitaxel: a clinical nomogram
Source: Front Pharmacol. 2026 Sep 8;17:1794696. doi: 10.3389/fphar.2026.1794696 (PMC13593910; doi:10.3389/fphar.2026.1794696)
Supplement: Supplementary file 1 [file Supplementaryfile1.docx]

Supplementary Material

**Table S1. Grading of CIPN Based on CTCAE v5.0**

| Grade | Definition | Clinical Features | Management Strategy |
| --- | --- | --- | --- |
| Grade 1 | Mild symptoms | Asymptomatic or mild paresthesia (e.g., numbness, tingling), no functional impairment | Continue treatment, observe; consider symptomatic management |
| Grade 2 | Moderate symptoms | Moderate sensory or motor symptoms; some interference with instrumental activities of daily living (ADL), self-care intact | Dose modification or symptomatic management; treatment usually continued |
| Grade 3 | Severe symptoms | Severe sensory or motor dysfunction; significant interference with self-care ADL (e.g., difficulty walking, holding objects, severe pain) | Interrupt treatment until recovery to ≤ grade 1; consider dose reduction upon resumption |
| Grade 4 | Life-threatening | Disabling neuropathy with life-threatening consequences; complete loss of function | Permanent discontinuation of causative agent; intensive supportive care |
| Grade 5 | Death | Death related to neurotoxicity | Not applicable |

Abbreviation: ADL, activities of daily living.

**Table S2. Baseline Characteristics and Intergroup Comparisons**

| Variables | Total (n = 403) | **CIPN < Grade 3** (n = 350) | **CIPN ≥ Grade 3** (n = 53) | Statistic | *P* |
| --- | --- | --- | --- | --- | --- |
|  |  |  |  |  |  |
| **Demographic characteristics** |  |  |  |  |  |
| Age, M (Q₁, Q₃) | 52.00  (45.00, 60.00) | 52.00  (45.25, 60.00) | 52.00  (45.00, 63.00) | Z=-0.27 | 0.790 |
| BMI, M (Q₁, Q₃) | 23.43  (22.04, 25.15) | 23.31  (22.03, 24.97) | 25.00  (22.98, 26.63) | Z=-3.14 | **0.002** |
| Menopause, n(%) |  |  |  | χ²=0.12 | 0.724 |
| No | 161 (39.95) | 141 (40.29) | 20 (37.74) |  |  |
| Yes | 242 (60.05) | 209 (59.71) | 33 (62.26) |  |  |
| Smoking, n(%) |  |  |  | χ²=16.10 | **< .001** |
| No | 367 (91.07) | 327 (93.43) | 40 (75.47) |  |  |
| Yes | 36 (8.93) | 23 (6.57) | 13 (24.53) |  |  |
| Alcohol drinking, n(%) |  |  |  | χ²=21.06 | **< .001** |
| No | 338 (83.87) | 305 (87.14) | 33 (62.26) |  |  |
| Yes | 65 (16.13) | 45 (12.86) | 20 (37.74) |  |  |
| Hypertension, n(%) |  |  |  | χ²=2.82 | 0.093 |
| No | 355 (88.09) | 312 (89.14) | 43 (81.13) |  |  |
| Yes | 48 (11.91) | 38 (10.86) | 10 (18.87) |  |  |
| Diabetes, n(%) |  |  |  | χ²=16.10 | **< .001** |
| No | 367 (91.07) | 327 (93.43) | 40 (75.47) |  |  |
| Yes | 36 (8.93) | 23 (6.57) | 13 (24.53) |  |  |
| **Tumor features** |  |  |  |  |  |
| Tumor Size, M (Q₁, Q₃) | 2.00 (2.00, 3.00) | 2.00 (2.00, 3.00) | 2.00 (2.00, 3.00) | Z=-1.05 | 0.294 |
| Tumor subtype, n(%) |  |  |  | χ²=5.81 | 0.121 |
| Luminal A | 109 (27.05) | 98 (28.00) | 11 (20.75) |  |  |
| Luminal B | 125 (31.02) | 103 (29.43) | 22 (41.51) |  |  |
| HER2+ | 80 (19.85) | 74 (21.14) | 6 (11.32) |  |  |
| TNBC | 89 (22.08) | 75 (21.43) | 14 (26.42) |  |  |
| Tumor stage, n(%) |  |  |  | - | 0.171 |
| I | 12 (2.98) | 10 (2.86) | 2 (3.77) |  |  |
| II | 266 (66.00) | 229 (65.43) | 37 (69.81) |  |  |
| III | 110 (27.30) | 100 (28.57) | 10 (18.87) |  |  |
| IV | 15 (3.72) | 11 (3.14) | 4 (7.55) |  |  |
| LN metastasis, n(%) |  |  |  | χ²=0.09 | 0.767 |
| No | 90 (22.33) | 79 (22.57) | 11 (20.75) |  |  |
| Yes | 313 (77.67) | 271 (77.43) | 42 (79.25) |  |  |
| Distant Metastasis, n(%) |  |  |  | χ²=1.41 | 0.234 |
| No | 388 (96.28) | 339 (96.86) | 49 (92.45) |  |  |
| Yes | 15 (3.72) | 11 (3.14) | 4 (7.55) |  |  |
| **Therapeutic parameters** |  |  |  |  |  |
| Treatment, n(%) |  |  |  | χ²=3.97 | 0.137 |
| Neo-adjuvant | 214 (53.10) | 191 (54.57) | 23 (43.40) |  |  |
| Adjuvant | 174 (43.18) | 148 (42.29) | 26 (49.06) |  |  |
| Palliative | 15 (3.72) | 11 (3.14) | 4 (7.55) |  |  |
| Chemotherapy dose, n(%) |  |  |  | χ²=0.00 | 0.989 |
| Conventional | 281 (69.73) | 244 (69.71) | 37 (69.81) |  |  |
| Dense | 122 (30.27) | 106 (30.29) | 16 (30.19) |  |  |
| **Laboratory tests** |  |  |  |  |  |
| TP, Mean ± SD | 71.80 ± 5.34 | 72.03 ± 5.25 | 70.27 ± 5.68 | t=2.25 | **0.025** |
| Blood glucose, M (Q₁, Q₃) | 5.38 (4.83, 6.08) | 5.39 (4.83, 6.08) | 5.33 (4.84, 5.90) | Z=-0.00 | 0.997 |
| Hb, Mean ± SD | 126.22 ± 13.50 | 126.83 ± 13.37 | 122.22 ± 13.84 | t=2.32 | **0.021** |
| AST, M (Q₁, Q₃) | 24.00 (19.00, 31.00) | 24.00 (19.00, 31.27) | 24.02 (18.00, 29.00) | Z=-0.61 | 0.539 |
| ALT, M (Q₁, Q₃) | 21.00 (15.57, 29.02) | 21.00 (15.63, 29.27) | 21.00 (15.53, 28.09) | Z=-0.00 | 0.997 |
| ALB, M (Q₁, Q₃) | 44.13 (41.21, 46.66) | 44.26 (41.28, 46.65) | 43.14 (40.54, 46.74) | Z=-0.82 | 0.414 |
| TBIL, M (Q₁, Q₃) | 10.61 (7.50, 13.67) | 10.44 (6.93, 13.25) | 12.27 (8.82, 14.56) | Z=-2.47 | **0.014** |
| DBIL, M (Q₁, Q₃) | 3.20 (2.20, 4.39) | 3.10 (2.18, 4.36) | 3.42 (2.57, 4.75) | Z=-1.37 | 0.172 |
| ALP, M (Q₁, Q₃) | 79.00 (62.73, 94.95) | 79.00 (62.34, 95.86) | 81.51 (64.81, 89.06) | Z=-0.01 | 0.994 |
| RBC, M (Q₁, Q₃) | 4.23 (3.96, 4.51) | 4.23 (3.96, 4.52) | 4.16 (3.90, 4.47) | Z=-0.73 | 0.463 |
| WBC, M (Q₁, Q₃) | 5.77 (4.80, 6.72) | 5.75 (4.79, 6.73) | 5.88 (4.91, 6.65) | Z=-0.71 | 0.479 |
| ANC, M (Q₁, Q₃) | 3.68 (2.97, 4.49) | 3.71 (3.00, 4.50) | 3.53 (2.85, 4.31) | Z=-0.81 | 0.419 |
| PLT, M (Q₁, Q₃) | 220.26 (182.12, 256.98) | 224.22 (188.00, 259.52) | 193.93 (162.61, 237.67) | Z=-3.11 | **0.002** |
| GFR, M (Q₁, Q₃) | 95.16 (83.66, 105.10) | 94.78 (83.90, 104.50) | 95.20 (82.52, 107.03) | Z=-0.26 | 0.796 |
| Cr, M (Q₁, Q₃) | 60.50 (49.88, 74.91) | 60.20 (49.80, 74.30) | 62.30 (52.50, 81.31) | Z=-0.90 | 0.368 |

Abbreviations: CIPN, chemotherapy-induced peripheral neuropathy; t: t-test, Z: Mann-Whitney test, χ²: Chi-square test, -: Fisher exact；SD: standard deviation, M: Median, Q₁: 1st Quartile, Q₃: 3st Quartile; LN, lymph node; TP, total protein; Hb, hemoglobin; AST, aspartate aminotransferase; ALT, alanine aminotransferase; ALB, albumin; TBIL, total bilirubin; DBIL, direct bilirubin; ALP, alkaline phosphatase; RBC, red blood cell count; WBC, white blood cell count; ANC, absolute neutrophil count; PLT, platelet count; GFR, glomerular filtration rate; Cr, creatinine.
